# UltRaSoUnd‐guided evaLuAtion of metabolic health in a cohort of midlife women with HIV: The URSULA study

**DOI:** 10.1111/hiv.70290

**Published:** 2026-07-28

**Authors:** Mariacristina Poliseno, Davide Capruzzi, Antonio Cianciaruso, Nicola Colavito, Felicia Dalitto, Vincenzo Giliberti, Vittoria Lobalsamo, Giuliana Metrangolo, Marica Noviello, Alessandra Vigna, Maurizio Zarcone, Francesco Di Gennaro, Vincenzo Ostilio Palmieri, Annalisa Saracino

**Affiliations:** ^1^ Clinic of Infectious Diseases, DiMePRe‐J University of Bari “Aldo Moro” Bari Italy; ^2^ Clinic of Internal Medicine, AOUC Policlinico di Bari Bari Italy; ^3^ Palermo and Province Cancer Registry, Clinical Epidemiology Unit with Cancer Registry, Azienda Ospedaliera Universitaria Policlinico “Paolo Giaccone”, University of Palermo Palermo Italy

**Keywords:** liver steatosis, metabolic syndrome, ultrasonography, visceral fat depot, women with HIV

## Abstract

**Objectives:**

We aimed to determine the prevalence of metabolic disorders, liver steatosis and metabolic dysfunction‐associated steatotic liver disease (MASLD) in a cohort of middle‐aged women with HIV (WoWH) and to explore associations between liver steatosis, visceral fat depots and carotid atherosclerosis. We also assessed the contribution of lifestyle, HIV‐ and antiretroviral therapy (ART)‐related factors to liver steatosis and the accuracy of United States National Cholesterol Education Program/Adult Treatment Panel III (NCEP–ATP III) criteria in identifying at‐risk patients.

**Methods:**

Between 1 April and 31 July 2025, all ART‐experienced WoWH aged ≥40 years received comprehensive metabolic and laboratory assessment, including abdominal and carotid Doppler ultrasound (US). Visceral fat depots were evaluated, and metabolic syndrome (MetS) was defined according to NCEP–ATP III criteria. Demographic, clinical and immunovirological data were collected. Logistic regression analyses identified factors associated with liver steatosis. Spearman correlations assessed associations between steatosis and fat distribution, and receiver operating characteristic (ROC) curves evaluated NCEP–ATP III accuracy in identifying liver steatosis.

**Results:**

Among 98 WoWH, 55 (56%) had at least grade I liver steatosis, 24 (25%) had MetS. Overall, MASLD was diagnosed in 41 participants (76%). Slight carotid intima‐media thickness (cIMT) increases were observed, and 35 participants (36%) had carotid plaques. MetS was the main risk factor for steatosis (OR 14.14, 95% C.I. 3.23–84.40, *p* = 0.001), with no HIV‐ or ART‐related associations. Progressive liver steatosis correlated with increasing retroperitoneal (rho = 0.393; *p* = 0.0001), subcutaneous (rho = 0.277; *p* = 0.005) and epicardial fat (rho = 0.312; *p* = 0.002) thickness. Conversely, epicardial fat thickness was not linked to cIMT. NCEP–ATP III criteria showed limited accuracy (AUC = 0.67) in identifying patients at risk for liver steatosis.

**Conclusions:**

In the URSULA cohort, hepatic steatosis—largely driven by MetS—was highly prevalent and associated with abnormal visceral fat depots. US may offer a cost‐effective tool for fast metabolic surveillance.

## INTRODUCTION

Over recent decades, an increasing incidence of chronic non‐communicable diseases (NCDs) has been observed among people with HIV, particularly metabolic syndrome (MetS), a cluster of interrelated risk factors that, according to the United States National Cholesterol Education Program/Adult Treatment Panel III (NCEP‐ATP III) criteria, includes at least three of the following features: abdominal obesity, low levels of high‐density lipoprotein cholesterol (HDL‐Chol), increased fasting glucose levels and hypertension [1, 2].

As a direct consequence of this trend, the prevalence of metabolic dysfunction‐associated steatotic liver disease (MASLD) is also high and rising among people with HIV. MASLD is increasingly recognized as a major driver of both morbidity and mortality in this population, contributing to cardiovascular and liver‐related comorbidities and exhibiting a more aggressive clinical trajectory than that observed in the general population [3, 4].

Multiple factors have been implicated in the development of metabolic complications among people with HIV, including HIV‐related chronic inflammatory stimuli associated with persistent immune activation, metabolic imbalances and end‐organ damage [5], as well as the metabolic perturbations observed in association with antiretroviral therapy (ART) [6]. These effects appear particularly evident in association with Integrase Strand Transfer Inhibitors (INSTIs), which have been associated with dyslipidaemia and weight gain [7].

Women with HIV (WoWH) are known to be more severely affected by metabolic perturbations compared with both women without HIV and men living with HIV, due to a complex interplay of biological, hormonal, cultural and social factors [6, 8]. Within this context, the menopausal transition has been associated with worsening of the metabolic profile of WoWH through paraphysiological metabolic perturbations related to declining ovarian oestrogen production. Oestrogens have also been shown to modulate the HIV latent reservoir and viral replication, suggesting that midlife may represent a particularly vulnerable period associated with higher rates of metabolic complications in this population [9].

Nevertheless, despite local differences related to socioeconomic contexts and healthcare system organization, WoWH remain a disadvantaged group not only in terms of higher rates of HIV transmission, delayed diagnosis and disease progression, but also regarding access to and utilization of high‐quality HIV care, beginning with access to ART [10]. Moreover, dedicated strategies and pathways for the prevention of NCDs in this population are either lacking or extremely limited.

Our analysis will investigate the metabolic health in a real‐life cohort of WoWH across pre‐ and post‐menopausal stages, with a particular focus on the contribution of traditional, HIV‐related and ART‐related factors to the development of liver steatosis with the aim of providing data that may support clinicians and policymakers in developing effective, integrated care networks for people with HIV, with particular attention to women, prioritizing the prevention of metabolism‐related disorders.

## MATERIALS AND METHODS

### Data collection and study population

The URSULA Study (UltRaSoUnd‐guided evaLuAtion of Metabolic Health in a Cohort of Midlife Women Living with HIV) is a single‐centre, cross‐sectional, observational analysis focusing on a cohort of WoWH aged ≥40 years and receiving ART from at least 6 months. The study involved the HIV Clinic and the Clinic of Internal Medicine of the Azienda Ospedaliero‐Universitaria Consorziale (AOUC) ‘Policlinico di Bari’ (Bari, Italy).

The main objectives of the study were:

- to determine the prevalence of metabolic disorders (including type 2 diabetes mellitus, dyslipidaemia, arterial hypertension, obesity and MetS) and, concurrently, to assess the prevalence and severity of liver steatosis and MASLD, the magnitude of visceral extrahepatic fat distribution and the presence and thickness of atherosclerotic plaques in the carotid arteries,
- to explore the association between liver steatosis, visceral fat distribution and cIMT, as well as between epicardial fat levels and cIMT and/or epicardial fat levels and the presence of atherosclerotic plaques,
- to identify the contribution of traditional lifestyle factors, HIV‐related factors and ART‐related factors to an increased risk of liver steatosis and
- to assess the accuracy of the NCEP–ATP III clinical criteria for MetS in identifying also patients at risk of liver steatosis.

To achieve these aims, between 1 April 1 and 31 July 2025, all WoWH aged >40 years who were followed at the HIV outpatient service of the Clinic of Infectious and Tropical Diseases of the Policlinic of Bari were offered a comprehensive clinical, laboratory and echographic assessment, including abdominal and carotid Doppler US.

We screened all WoWH who were on active follow‐up during the 12 months preceding study enrolment (defined as at least one clinical visit or laboratory test performed within the aforementioned time span), had been receiving stable ART for at least 6 months regardless of virological suppression status and were able to understand and provide written informed consent.

Participants presenting with acute intercurrent conditions at enrolment that could interfere with the validity of laboratory or instrumental assessments, or with pre‐existing metabolic disorders diagnosed prior to HIV infection, were excluded. Conversely, WoWH co‐infected with hepatitis B virus (HBV) and/or hepatitis C virus (HCV) were included, as these conditions represent clinically relevant comorbidities and potential additional risk factors for liver disease. Similarly, patients declaring alcohol consumption exceeding MASLD thresholds were included in the analysis. Alcohol intake was assessed descriptively and quantified as more than one standard drink per day (i.e., >10–12 g of pure ethanol per day, as self‐reported by patients).

Patient selection is illustrated in the flowchart in Figure 1.

**FIGURE 1:**
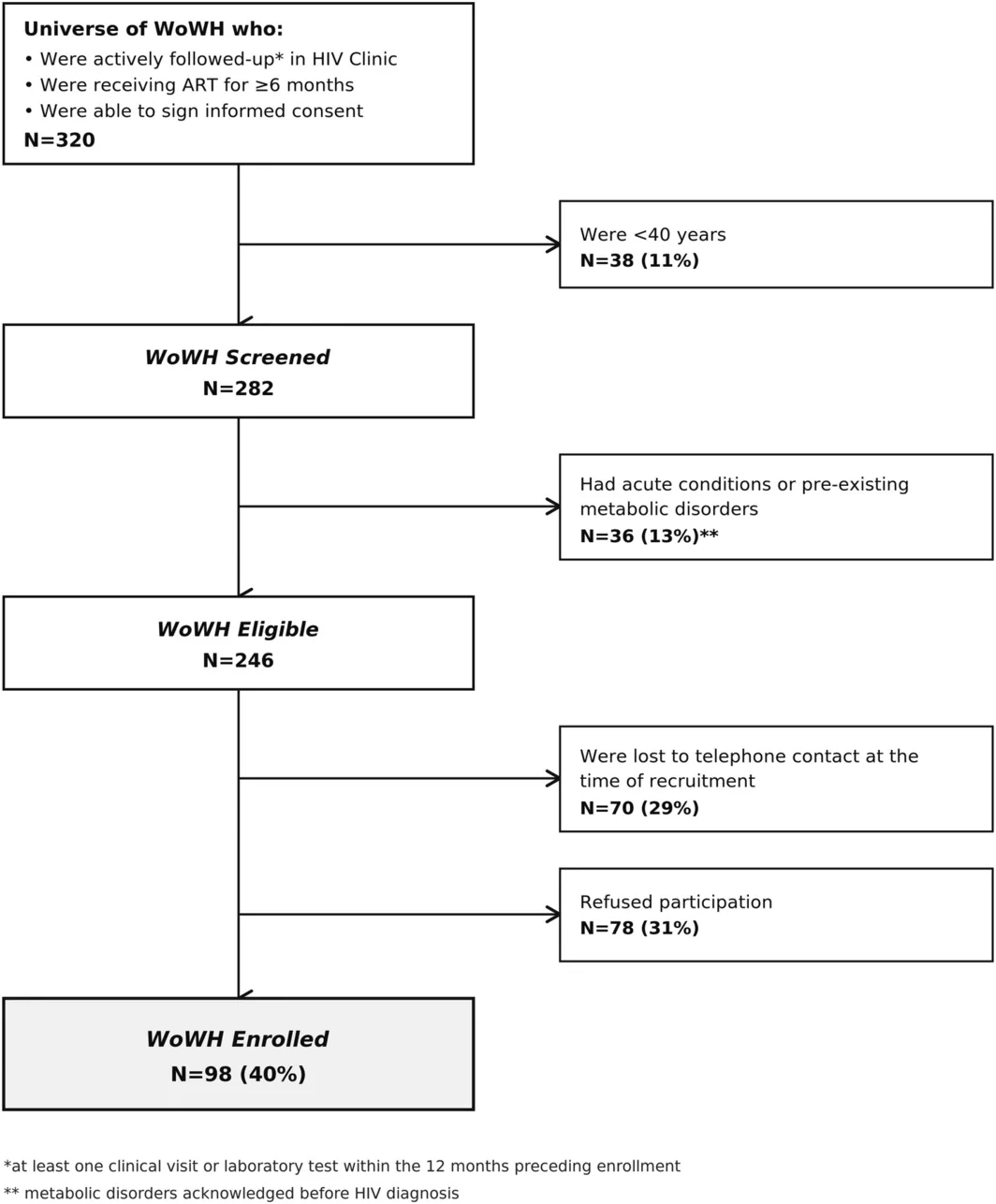
The diagram illustrates the screening and inclusion process of WoWH aged >40 years attending the outpatient clinic, the application of eligibility criteria, exclusions and the final study population included in the analysis.

All WoWH underwent a comprehensive medical evaluation, including collection of personal and family medical history, lifestyle habits (occupation, diet, physical activity, smoking status, alcohol consumption and substance use) and measurement of anthropometric parameters (weight, height, waist circumference, arterial blood pressure and heart rate). Gynaecological history was also collected, with particular attention to menopausal status, defined as pre‐menopausal or post‐menopausal based on the presence or absence of menstruation for less than or more than 12 months, respectively.

A predefined laboratory panel was performed, including total cholesterol (T‐Chol, <200 mg/dL), low‐density lipoprotein cholesterol (LDL‐Chol, <110 mg/dL), HDL‐Chol (>50 mg/dL), triglycerides (TGL, <150 mg/dL), aspartate aminotransferase (AST, <34 U/L), alanine aminotransferase (ALT, <50 U/L), Chronic Kidney Disease Epidemiology Collaboration (CKD‐EPI) estimated glomerular filtration rate (eGFR, >90 mL/min/1.73 m^2^), sodium (135–145 mmol/L), potassium (3.5–5.0 mmol/L), blood urea nitrogen (BUN, 7–20 mg/dL), uric acid (2.4–5.7 mg/dL), ammonia (15–45 μg/dL), D‐dimer (<500 ng/mL FEU), C‐reactive protein (CRP, <4 mg/dL), iron (60–170 μg/dL), ferritin (15–150 ng/mL), transferrin (200–360 mg/dL), fasting glucose (<100 mg/dL), insulin (2–25 μIU/mL), haemoglobin A1c (HbA1c, <5.7%), thyroid‐stimulating hormone (TSH, 0.4–4.0 mIU/L), free thyroxine (fT4, 0.8–1.8 ng/dL) and free triiodothyronine (fT3, 2.3–4.2 pg/mL). Reference values for these variables were defined according to international guidelines. [11, 12, 13].

Based on the available data, the Framingham 10‐year risk score for hard coronary heart disease (HCHD) was calculated for all eligible participants [14]. Participants were also screened for MetS according to the NCEP–ATP III criteria and classified as having MetS in the presence of ≥3 diagnostic components or if receiving pharmacological treatment for related conditions (e.g., antihypertensive, glucose‐lowering or lipid‐lowering medications) [1]. Body mass index (BMI) was calculated using the formula weight (kg)/height (m)^2^.

Each week, two dedicated days were scheduled at the Clinic of Internal Medicine, during which enrolled WoWH underwent a comprehensive US evaluation, including: (i) assessment of the liver [volume (pre‐aortic, mid‐clavicular and pre‐renal diameters), parenchymal echotexture, attenuation, vascular pattern, liver stiffness by elastography, grading of steatosis (Grade 0–3) and detection of focal lesions or accessory findings]; (ii) assessment of the portal system [measurement of portal and splenic vein diameters, Doppler flow evaluation of the portal and paraumbilical veins and identification of thrombotic or cavernomatous changes]; (iii) morphological and structural assessment of the gallbladder, pancreas, spleen and kidneys; (iv) evaluation of the supra‐aortic trunks (measurement of cIMT and detection of plaques or stenoses with quantification); (v) visceral fat distribution (quantification of abdominal, pre‐peritoneal, subcutaneous, epicardial and perirenal fat).

For diffuse liver tissue elastography, we used an US system (Acuson S2000, Siemens Healthcare Srl, Forchheim, Germany) equipped with point Shear Wave Elastography (pSWE) technology, a shear wave elastometry technique in which shear wave velocity generated by acoustic radiation force impulse is measured within a precise region of interest (ROI) in the liver parenchyma. The standard protocol included measurements in the right liver lobe (lateral sector), approximately 5 cm below the liver capsule, in fasting patients, with repeated acquisitions across at least 10 different ROIs. Final results were expressed as mean ± standard deviation (M ± SD).

Hepatic steatosis was assessed by conventional ultrasonography using the same equipment and a 3.5 MHz convex probe. Renal cortex echogenicity was used as an internal reference for liver parenchymal echogenicity, allowing classification of the liver as isoechoic (normal) or hyperechoic (‘bright liver’) in the presence of steatosis. Steatosis severity was graded from 0 to 3: Grade 0 (absent, normal liver echotexture), Grade 1 (mild, slight diffuse increase in echogenicity), Grade 2 (moderate, marked increase in echogenicity with impaired visualization of the posterior segments of the right lobe) and Grade 3 (severe, marked increase in echogenicity with poor or non‐visualization of intrahepatic vessels and diaphragm).

Liver stiffness was also assessed as part of the clinical and instrumental evaluation to exclude the presence of advanced hepatic fibrosis in the study population. As liver stiffness is a validated surrogate marker of hepatic fibrosis rather than steatosis, it was not included as an outcome or predictor in the statistical analyses but was instead used to exclude clinically relevant fibrosis as a potential confounding factor, ensuring that all liver‐related findings in our cohort could be attributed to steatosis rather than fibrosis.

MASLD was diagnosed in the presence of at least Grade 1 hepatic steatosis plus at least one of the following cardiometabolic risk factors: (i) arterial blood pressure ≥130/80 mmHg or antihypertensive treatment; (ii) fasting blood glucose >100 mg/dL or glucose‐lowering treatment; (iii) abdominal circumference >88 cm; (iv) HDL‐C < 50 mg/dL; and (v) TGL >150 mg/dL or lipid‐lowering treatment.

Finally, baseline demographic and immunovirological data—including year and route of HIV transmission, CD4 cell nadir, HIV RNA zenith, HCV and/or HBsAg positivity, and the presence of AIDS‐defining conditions at baseline—were retrospectively extracted from electronic medical records (NetCare®, Healthware, Salerno, Italy) and analysed anonymously. Special attention was paid to the type of ART regimen prescribed at the time of the study and to cumulative exposure to third‐drug classes associated with potential adverse metabolic effects, such as tenofovir alafenamide (TAF), protease inhibitors (PIs) and INSTIs. AIDS‐defining conditions were classified according to the 1993 revised Centers for Disease Control and Prevention (CDC) criteria for HIV infection [15].

### Statistical analysis

Population characteristics were described using frequencies and percentages for categorical variables and means (± standard deviation, SD) or medians (interquartile range, IQR) for continuous variables, according to their parametric or non‐parametric distribution as assessed by normality tests. Differences between WoWH with and without at least Grade I liver steatosis were evaluated using permutation tests (10 000 Monte Carlo iterations) for continuous variables and the χ^2^ test or Fisher's exact test, as appropriate, for categorical variables. Summary tables for the overall cohort and comparison groups, including corresponding *p* values, were generated using the *compare Groups* package. To account for multiple testing, *p* values were adjusted using the Benjamini–Hochberg procedure with a false discovery rate (FDR) set at 0.05 [16]. Categories with low frequency were collapsed or excluded to ensure model stability.

The association between liver steatosis and abnormal fat tissue distribution was assessed using Spearman's rank correlation to evaluate monotonic relationships and permutation tests (10 000 iterations) to compare mean values of subcutaneous, pre‐peritoneal and epicardial fat between participants with and without liver steatosis. The same analytical approach was used to assess, conversely, the association between mean epicardial fat values and mean body fat values measured at other anatomical sites, including the liver.

Associations between covariates and the presence of liver steatosis were expressed as odds ratios (ORs) with 95% confidence intervals (95% CIs) and corresponding *p* values. Multivariable logistic regression analyses were used to estimate adjusted associations. Covariates were selected a priori based on existing literature and biological plausibility, avoiding inclusion of highly collinear variables. The absence of multicollinearity (variance inflation factor >10) and perfect separation was verified. Categorical variables were coded as factors with predefined reference categories.

Finally, the discriminative performance of the NCEP–ATP III criteria for identifying liver steatosis was evaluated using receiver operating characteristic (ROC) curves, with estimation of the area under the curve (AUC) and corresponding 95% CIs. Comparisons between ROC curves were performed using the DeLong test [17]. Model parsimony was optimized through bidirectional stepwise selection based on the Akaike information criterion (AIC) [18], implemented using the *stepAIC* function from the MASS package.

Data were managed and analysed using the R® statistical environment (version 4.2.2). The Microsoft Excel® (.xlsx) dataset was imported using the *readxl* package. Statistical significance was set at *p* < 0.05.

## RESULTS

Overall, 246 eligible WoWh were identified, of whom 98 consented to participate in the study.

Of these, 43 (44%) had no evidence of liver steatosis (women with HIV w/o steatosis), while 55 (56%) presented with at least Grade 1 liver steatosis. Among participants with steatosis, 38 (69%) had Grade 1, 16 (29%) Grade 2 and 1 (2%) Grade 3 disease. Few differences, mainly related to lifestyle factors and medical history, were observed between the two groups, which were otherwise comparable in terms of age (mean 57 ± 7 vs. 55 ± 7 years, *p* = 0.24) and prevalence of women with HIV in menopause (35, 64% vs. 32, 74%; *p* = 0.45). The main characteristics of the URSULA cohort are reported in Table 1.

**TABLE 1:** General baseline features of 98 women with HIV (WoWH) in the URSULA cohort.

Variables	Overall (*N* = 98)	WoWH w/o steatosis (*N* = 43)	WoWH with steatosis (*N* = 55)	*p* value*
Age, years, mean (± SD)	56 (±7)	55 (±7)	57 (±7)	0.24
Age range, *n* (%)				
≥ 60 years	28 (29)	10 (23)	18 (33)	0.31
≥ 55 years	62 (63)	26 (60)	36 (65)	0.62
≥ 50 years	78 (80)	33 (77)	45 (82)	0.55
Menopause, *n* (%)	67 (68)	32 (74)	35 (64)	0.45
Menopause duration, years, mean (± SD)	6 (±6)	5 (±5)	7 (±7)	0.13
Lifestyle habits, *n* (%)				
Smoking or smoking history	51 (52)	23 (54)	28 (51)	0.87
Physical activity	22 (22)	10 (23)	12 (22)	0.99
Alcohol consumption**	7 (7)	1 (2)	6 (11)	0.09
Work activity, *n* (%)	45 (46)	24 (56)	21 (38)	0.12
Arduous work***, *n* (%)	26 (27)	13 (30)	13 (24)	0.47
Duration of work activity, years, mean (± SD)	23 (±11)	20 (±9)	27 (±11)	**0.044**
Family medical history, *n* (%)				
Dyslipidaemia	46 (47)	16 (37)	30 (55)	0.06
Cardiac events	24 (25)	7 (16)	17 (31)	0.11
Type II diabetes mellitus	19 (19)	8 (19)	11 (20)	0.86
Obesity	10 (10)	2 (5)	8 (15)	0.11
Stroke	9 (9)	2 (5)	7 (13)	0.18
Renal failure	7 (7)	4 (9)	3 (5)	0.50
Comorbidities, *n* (%)				
Dyslipidaemia	49 (50)	18 (42)	31 (56)	0.16
Arterial hypertension	33 (34)	8 (19)	25 (46)	**0.006**
Cardiovascular diseases	8 (8)	1 (2)	7 (13)	0.07
Type II diabetes mellitus	7 (7)	2 (5)	5 (9)	0.44
Obesity	6 (6)	2 (5)	4 (7)	0.63
Renal failure	5 (5)	3 (7)	2 (4)	0.50
History of cardiac infarction	3 (3)	0 (0)	3 (5)	0.99
Cerebrovascular diseases	2 (2)	0 (0)	2 (4)	0.99
Gout	2 (2)	0 (0)	2 (4)	0.99
Use of statins, *n* (%)	33 (34)	8 (19)	25 (46)	**0.006**
≥2 comedications, *n* (%)	43 (44)	13 (30)	30 (55)	**0.018**

*Note:* Bold indicates significant *p*‐values.

Abbreviations: AMI, acute myocardial infarction; BMI, body mass index; CKD, chronic kidney disease; DM, diabetes mellitus; IQR, interquartile range; MetS, metabolic syndrome.

* Chi‐square test/Student's *t*‐test as appropriate.

** More than 1 standard drink per day (i.e., >10–12 g of pure ethanol per day) as reported by patients.

*** Farm labourer, domestic worker, factory labourer, healthcare assistant, nurse, factory worker, hotel/hospital cleaner.

Participants had a long history of HIV infection and ART, with no significant differences according to the presence of liver steatosis. Four WoWH (4%) had HBV coinfection and 17 (17%) were HCV antibody–positive; all had undetectable HCV RNA levels.

At the time of the study, a large proportion of participants were receiving an INSTI‐based regimen (81 women with HIV, 82%), either as two‐drug combinations (33 women with HIV, 34%) or three‐drug regimens. However, a substantial number of participants (66, 67%) had been exposed to PI‐based regimens during their treatment history, for a median duration of 7 (±7) years. Similarly, no differences were observed between the two study groups.

Immunovirological and antiretroviral treatment characteristics of the study population are reported in Table 2.

**TABLE 2:** Immunovirological and antiretroviral therapeutic features of study population.

Variables	Overall (*N* = 98)	WoWH w/o steatosis (*N* = 43)	WoWH with steatosis (*N* = 55)	*p* value*
HIV duration, years, mean (± SD)	25 (±10)	26 (±9)	24 (±11)	0.25
AIDS diagnosis at baseline, *n* (%)	17 (18)	6 (14)	11 (21)	0.43
HBsAg positive, *n* (%)	4 (4)	2 (5)	2 (4)	0.83
HCV‐Ab positive, *n* (%)	17 (17)	8 (19)	9 (16)	0.78
CD4 cell count nadir, cell/mm^3^, mean (± SD)	208 (±141)	207 (±148)	208 (±136)	0.95
HIV RNA zenith, log_10_(cp/mL), mean (± SD)	5 (±6)	6 (±5)	5 (± 6)	0.81
Undetectable VL, *n* (%)	74 (76)	31 (72)	43 (78)	0.50
ART duration, years, mean (± SD)	21 (±9)	21 (±9)	21 (±10)	0.88
ART regimens, *n* (%)				0.72
NNRTI‐based	9 (16)	10 (22)	9 (16)	
PI‐based	8 (8)	3 (7)	5 (9)	
INSTI‐based	73 (73)	32 (71)	41 (74)	
Currently on STR, *n* (%)	72 (74)	31 (72)	41 (75)	0.79
Currently on LA regimen, *n* (%)	9 (9)	5 (12)	4 (7)	0.48
Current use of TAF, *n* (%)	35 (36)	16 (37)	19 (35)	0.79
History of INSTIs use, *n* (%)	86 (88)	36 (84)	50 (91)	0.30
INSTIs use duration, years, mean (± SD)	6 (4)	5 (±4)	6 (±4)	0.34
History of TAF use, *n* (%)	65 (66)	32 (74)	33 (60)	0.05
TAF use duration, years, mean (± SD)	3 (3)	4 (±3)	3 (±3)	0.08
History of PIs use, *n* (%)	66 (67)	30 (70)	36 (66)	0.66
PIs use duration, years, mean (± SD)	7 (7)	7 (±7)	7 (±7)	0.82

Abbreviation: AIDS, acquired immunodeficiency syndrome; ART, antiretroviral therapy; HBsAg, hepatitis B surface antigen; HCV‐Ab, hepatitis C antibodies; HIV, human immunodeficiency virus; INSTI, integrase strand transfer inhibitor; LA, long‐acting; NNRTI, non‐nucleoside reverse transcriptase inhibitor; NRTI, nucleoside reverse transcriptase inhibitor; PI, protease inhibitor; SD, standard deviation; STR, single tablet regimen; TAF, tenofovir alafenamide fumarate; VL, viral load; WoWH, women with HIV.

* Chi‐square test/Student's *t*‐test as appropriate.

MetS was diagnosed in 24 WoWH overall (25%) and was significantly more prevalent among those with hepatic steatosis than among those without steatosis (40% vs. 7%, *p* < 0.001).

Indeed, MASLD was identified in 41 of the 55 WoWH with hepatic steatosis (76.4%). Specifically, 21 participants had hepatic steatosis in combination with three metabolic risk factors, 8 had hepatic steatosis in combination with two metabolic risk factors, and 12 had hepatic steatosis and only one metabolic risk factor. The remaining 13 women with HIV had isolated hepatic steatosis in the absence of metabolic risk factors.

At metabolic laboratory assessment, as expected, WoWH with liver steatosis showed higher mean fasting glucose (91 vs. 84 mg/dL, *p* = 0.025) and fasting insulin levels (16 vs. 11 mIU/L, *p* = 0.016) and lower HDL‐Chol levels (55 vs. 63 mg/dL, *p* = 0.011) than those without steatosis. No differences were observed in renal or thyroid function, liver cytolysis indices, iron status, TGL or inflammatory markers (D‐dimers and CRP). Detailed data are reported in Table S1.

Dyslipidaemia and arterial hypertension were the most represented metabolic comorbidities, the latter of which was highly prevalent in the group of WoWH with liver steatosis (46 vs. 19%, *p* = 0.006). Participants with liver steatosis also had a higher waist circumference (91 vs. 84 cm, *p* = 0.014) and a higher prevalence of waist circumference ≥88 cm (59% vs. 32%, *p* = 0.013), fasting glucose ≥100 mg/dL (22% vs. 5%, *p* = 0.016) and arterial blood pressure ≥130/80 mmHg at clinical visit (56% vs. 33%, *p* = 0.026). No differences were observed in weight, height or BMI (Table S2).

At abdominal and carotid Doppler US assessment, apart from the different prevalence of liver steatosis, no significant differences were found in liver and spleen dimensions or stiffness, nor in collateral venous circulation. Carotid artery plaques were detected in 35 women with HIV (36%), of whom 12 (12% of total population) initiated a new antiplatelet and/or lipid‐lowering therapy. As expected, WoWH with liver steatosis showed higher mean abdominal (34.3 vs. 25 mm, *p* = 0.005), subcutaneous (14.5 vs. 12.3 mm, *p* = 0.033), epicardial (5.62 vs. 4.71 mm, *p* = 0.018) and right perirenal visceral (4.79 vs. 3.72 mm, *p* = 0.005) fat thicknesses. Detailed US findings are reported in Table 3.

**TABLE 3:** Ultrasound findings in the overall population and in the women with HIV (WoWH) with and without steatosis subpopulations.

Ultrasound findings	Overall (*N* = 98)	WoWH w/o steatosis (*N* = 43)	WoWH with steatosis (*N* = 55)	*p* value*
Liver				
Diameter, mm, mean (± SD)				
Pre‐aortic	84.1 (±20.3)	82.4 (±18.6)	85.5 (±21.6)	0.46
Hemiclavicular	109 (±19.6)	106 (±15.6)	112 (±22.1)	0.17
Pre‐renal	137 (±19.8)	135 (±18.5)	139 (±20.8)	0.27
Grade of steatosis, *n* (%)				
No steatosis	43 (44)	43 (100)	0 (0)	/
Grade 1	38 (39)	0 (0)	38 (69)	/
Grade 2	16 (16)	0 (0)	16 (29)	/
Grade 3	1 (1)	0 (0)	1 (2)	/
Stiffness, m/s, mean (± SD)	1.14 (±0.37)	1.07 (±0.17)	1.20 (±0.47)	0.13
Presence of right hepatic vein triphasic flow pattern, *n* (%)	59 (62)	24 (57)	35 (66)	0.39
Hepatic vein calibre, mm, mean (± SD)	6.19 (±1.94)	6.43 (±2.13)	6.01 (±1.78)	0.30
Portal vein calibre, mm, mean (± SD)	9.21 (±1.50)	8.88 (±1.62)	9.46 (±1.36)	0.06
Portal vein flow, cm/s, mean (± SD)	30.7 (±10.2)	30.7 (±10.8)	30.8 (±9.83)	0.99
Spleen				
Length, mm, mean (± SD)	89.9 (28.2)	89.2 (27.6)	90.4 (28.9)	0.84
Width, mm, mean (± SD)	45.7 (24.1)	45.3 (23.4)	46.0 (24.7)	0.89
Area, cm^2^, mean (± SD)	38.2 (55.2)	32.2 (10.1)	42.6 (72.3)	0.56
Stiffness, m/s, mean (± SD)	3.47 (2.37)	3.50 (3.23)	3.44 (1.39)	0.92
Splenic vein calibre, mm, mean (± SD)	5.74 (3.85)	5.45 (1.63)	5.97 (4.94)	0.53
Carotid artery				
Intima thickness, mm, mean (± SD)	0.79 (0.19)	0.78 (0.17)	0.80 (0.21)	0.63
Media thickness, mm, mean (± SD)	0.83 (0.25)	0.82 (0.25)	0.84 (0.25)	0.66
Presence of plaque, *n* (%)	35 (36)	17 (40)	18 (33)	0.49
New need of therapy, *n* (%)	12 (12)	6 (14)	6 (11)	0.66
Extrahepatic fat depot thickness				
Abdominal, mm, mean (± SD)	30.2 (15.7)	25.0 (11.8)	34.3 (17.1)	**0.005**
Pre‐peritoneal visceral fat, mm, mean (± SD)	13.4 (5.61)	12.2 (4.51)	14.4 (6.23)	0.06
Subcutaneous fat, mm, mean (± SD)	13.5 (4.93)	12.3 (4.14)	14.5 (5.31)	**0.033**
Epicardial fat, mm, mean (± SD)	5.21 (1.82)	4.71 (1.74)	5.62 (1.79)	**0.018**
Right perirenal visceral fat, mm, mean (± SD)	4.32 (1.78)	3.72 (1.29)	4.79 (1.97)	**0.005**
Left perirenal visceral fat, mm, mean (± SD)	4.95 (2.13)	4.65 (2.08)	5.19 (2.16)	0.22

*Note:* Bold indicates significant *p*‐values.

Abbreviation: SD, standard deviation; WoWH, women with HIV.

* Chi‐square test/Student's *t*‐test as appropriate.

At metabolic laboratory assessment, WoWH with liver steatosis showed higher mean fasting glucose (91 vs. 84 mg/dL, *p* = 0.025) and fasting insulin levels (16 vs. 11 mIU/L, *p* = 0.016) and lower HDL‐Chol levels (55 vs. 63 mg/dL, *p* = 0.011) than those without steatosis. No differences were observed in renal or thyroid function, liver cytolysis indices, iron status, TGL or inflammatory markers (D‐dimers and CRP). Detailed data are reported in Table S1.

A positive association between progressively higher grades of liver steatosis and increased adipose tissue accumulation across the different anatomical compartments examined was observed. Specifically, significant correlations were found for subcutaneous (ρ = 27.7, *p* = 0.0055), pre‐peritoneal (ρ = 39.3, *p* = 0.0246) and epicardial fat depots (ρ = 31.2, *p* = 0.0019).

Conversely, Spearman's correlation analysis of epicardial and other extrahepatic fat depot thicknesses revealed a significant positive correlation between epicardial fat and both right (ρ = 32.1, *p* = 0.010) and left perirenal fat (ρ = 35.8, *p* < 0.001), whereas no statistically significant correlations were observed with right or left cIMT or with other visceral fat depots.

It is worth mentioning that, following US assessment, incidental findings were reported in five participants. Specifically, these included one case of severe hydronephrosis, one case of portal cavernoma associated with splenomegaly, one case of hepatic space‐occupying lesions, one case of diffuse lymphadenopathy with heterogeneous thyroid alterations in a patient with a history of previous malignancy and one case of a left breast lesion in a patient under follow‐up for prior breast cancer. All cases subsequently underwent second‐level imaging investigations.

Logistic regression analyses were performed to identify potential protective and risk factors for the development of liver steatosis among women with HIV (Table 4). MetS was consistently associated with a substantially higher risk of hepatic steatosis in both crude and adjusted models (OR 10.00, 95% CI 3.08–45.28, *p* = 0.001; and OR 14.14, 95% CI 3.23–84.40, *p* = 0.001, respectively). In this cohort, no statistically significant association was observed between hepatic steatosis and HIV duration, ART duration or cumulative exposure to INSTIs or PIs.

**TABLE 4:** Univariable and multivariable logistic regression analyses assessing the relative risk of liver steatosis according to characteristics of women with HIV.

Covariates	Univariable O.R. (95% C.I., *p* value)	Multivariable O.R. (95% C.I., *p* value)
Age ≥60 years	1.92 (0.78–4.92, *p* = 0.16)	0.91 (0.21–3.54, *p* = 0.89)
Menopause	0.84 (0.32–2.16, *p* = 0.72)	0.45 (0.12–1.67, *p* = 0.24)
Arduous work	0.75 (0.30–1.84, *p* = 0.53)	0.59 (0.18–1.87, *p* = 0.38)
Physical activity	0.96 (0.36–2.57, *p* = 0.93)	0.79 (0.23–2.64, *p* = 0.70)
Current/former smoker	0.98 (0.43–2.24, *p* = 0.97)	0.78 (0.28–2.15, *p* = 0.63)
Alcohol consumption	5.86 (0.95–113.19, *p* = 0.11)	11.96 (1.04–356.55, *p* = 0.08)
Dyslipidaemia	2.25 (0.98–5.28, *p* = 0.06)	2.10 (0.68–6.65, *p* = 0.20)
BMI >25 kg/m^2^	1.83 (0.79–4.35, *p* = 0.16)	1.18 (0.39–3.53, *p* = 0.76)
MetS	10.00 (3.08–45.28, *p* = 0.001)	14.14 (3.23–84.40, *p* = 0.001)
≥10 years HIV	0.55 (0.07–2.97, *p* = 0.50)	0.54 (0.03–7.91, *p* = 0.65)
Nadir CD4 < 200 cell/mm3	0.91 (0.40–2.06, *p* = 0.81)	1.26 (0.41–4.02, *p* = 0.68)
≥10 years ART	0.76 (0.25–2.19, *p* = 0.61)	0.77 (0.16–3.75, *p* = 0.75)
≥10 years PI	0.91 (0.38–2.22, *p* = 0.84)	0.54 (0.13–1.99, *p* = 0.37)
≥10 years INSTI	1.06 (0.30–3.94, *p* = 0.93)	0.72 (0.12–4.15, *p* = 0.71)

Abbreviations: ART, antiretroviral therapy, BMI, body mass index; C.I., confidence interval; HIV, human immunodeficiency virus; INSTI, integrase strand transfer inhibitor; NNRTI, non‐nucleoside reverse transcriptase inhibitor; O.R., odds ratio; PI, protease inhibitor; STR, single tablet regimen.

To evaluate whether MetS diagnostic criteria alone could predict the presence of hepatic steatosis in the absence of US assessment, ROC curve analysis was performed. While the NCEP–ATP III criteria proved highly effective for the clinical and laboratory identification of MetS, their ability to discriminate the presence of liver steatosis, at least in our cohort, was not excellent (AUC of 0.67 (95% CI: 0.589–0.745)). (Figure 2).

**FIGURE 2:**
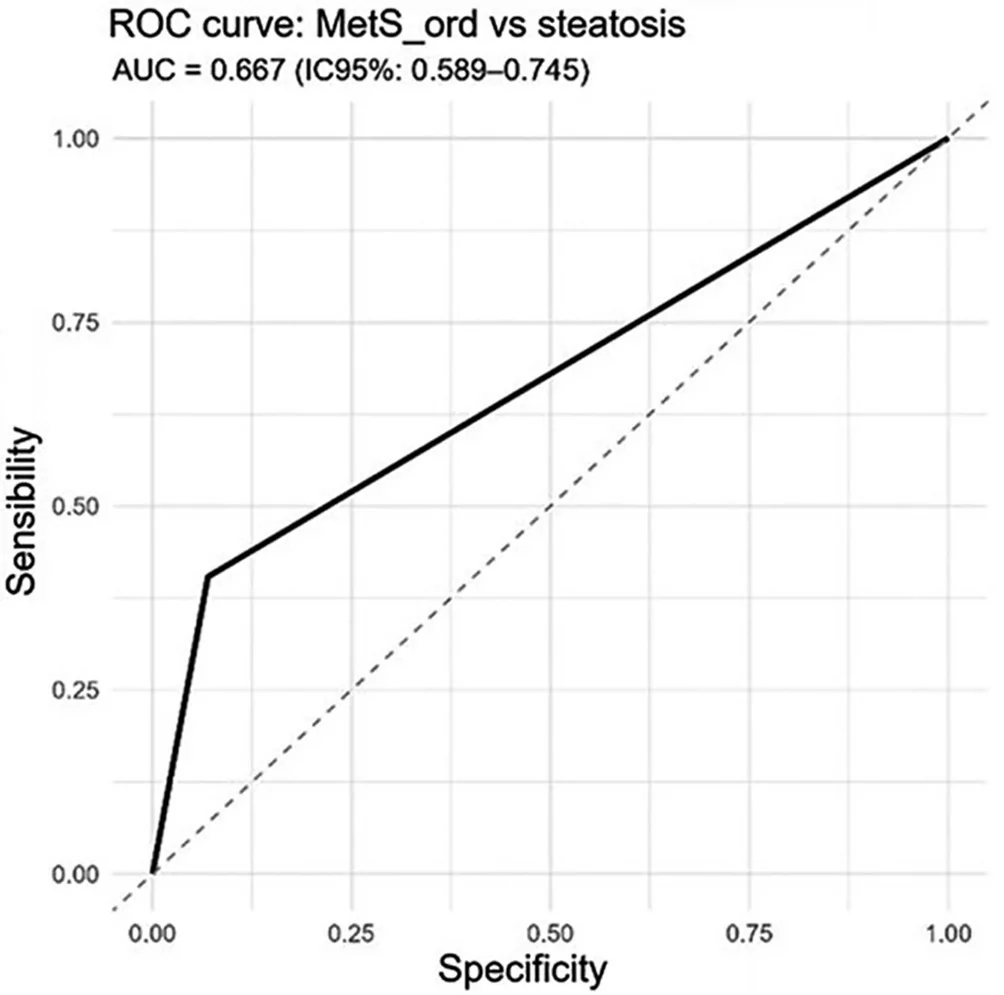
Receiver operating characteristic (ROC) curve illustrating the predictive performance of the NCEP–ATP III diagnostic criteria for metabolic syndrome (MetS) in identifying liver steatosis among women with HIV in the URSULA cohort. The cut‐off value was set at 0.5.

## DISCUSSION

The URSULA study aimed to address a relevant gap in current evidence regarding liver and metabolic health in midlife WoWH, a population underrepresented in clinical research.

We detected a high prevalence of hepatic steatosis, predominantly Grade 1, consistent with data reported in the literature for the general population of comparable age (approximately 56 years) and both sexes [19].

A notably high proportion of MetS cases (25%) was also observed, with elevated blood pressure and reduced HDL cholesterol levels representing the most frequent diagnostic criteria. Recent meta‐analyses in people with HIV estimate an overall MetS prevalence of 25.3%, corresponding to a 1.6‐fold higher risk compared to HIV‐seronegative individuals [2]. Specifically among WoWH, the prospective Women's Interagency HIV Study (WIHS) reported an even higher prevalence (33%), significantly exceeding that observed in HIV‐seronegative controls (22%). Dyslipidaemia, older age, higher BMI, tobacco use, detectable viraemia and exposure to older‐generation ART were identified as key determinants [20].

MASLD was diagnosed in 41 of the 55 women with HIV with hepatic steatosis, yielding a prevalence of 76% within this subgroup and an overall prevalence of 41% across the entire cohort. As expected, MetS emerged as the sole factor independently associated with a 7‐ to 10‐fold higher odds of hepatic steatosis in both unadjusted and adjusted analyses.

This finding aligns with the well‐established association between MetS and the progression of hepatic steatosis towards steatohepatitis, fibrosis and cirrhosis—a relationship that has recently prompted a revision of disease nomenclature, replacing non‐alcoholic fatty liver disease (NAFLD) and non‐alcoholic steatohepatitis (NASH) with the concepts of MASLD and metabolic dysfunction‐associated steatohepatitis (MASH), thereby placing metabolic dysfunction at the centre of liver disease pathogenesis [3, 21]. The reported prevalence of MASLD among people with HIV ranges from 20% to 63%, with 14%–63% of affected individuals presenting with MASH and fibrosis [3, 4], a range consistent with the findings of the URSULA cohort.

This result was not unexpected, given that people with HIV are at heightened risk for MASH and fibrosis and that recent studies have demonstrated an accelerated increase in hepatic risk scores among women with HIV aged 40–50 years—corresponding to the perimenopausal transition [19, 22]—a phase comparable to the median age of our cohort.

Although all participants were older than 40 years, we stratified the cohort by menopausal status and age class. Nevertheless, the prevalence of hepatic steatosis was comparable across these subgroups. Moreover, neither menopausal status nor chronological age, nor HIV‐specific factors—including HIV duration, baseline CD4 cell count, ART duration and cumulative exposure to PIs or INSTIs—independently predicted hepatic steatosis. MetS emerged as the sole robust predictor (adjusted OR 14.14, 95% CI 3.23–84.40, *p* = 0.001).

Rather than representing a methodological limitation, this finding may carry independent scientific significance. A possible explanation for these null associations is that oestrogen deficiency may exert its hepatosteatogenic effect primarily through its metabolic sequelae– visceral fat redistribution, insulin resistance and atherogenic dyslipidaemia– which are already captured by the MetS composite variable, rather than operating as an independent pathophysiological pathway. This interpretation is further supported by the high degree of virological suppression in our cohort (76% with undetectable viral load), which may have homogenized the metabolic background across menopausal subgroups.

Regarding the absence of a statistically significant association between cumulative exposure to contemporary ART classes and hepatic steatosis, it should be acknowledged that the limited sample size may have reduced the statistical power to detect associations of small to moderate effect size.

Furthermore, the literature on TAF‐ and INSTI‐based regimens remains limited and inconsistent. Large cohort studies in mixed‐sex people with HIV have associated hepatic steatosis primarily with traditional metabolic risk factors (age, BMI, overweight/obesity) rather than ART‐specific variables [23]. In contrast, the Swiss HIV Cohort Study reported associations with BMI ≥25 kg/m^2^, age ≥50 years and current TAF use [24]. Analyses from the WIHS and the multisite prospective liver disease and reproductive ageing study indicate that ART regimen, CD4 cell count and viral load were not significantly associated with controlled attenuation parameter (CAP)‐assessed hepatic steatosis in women with HIV, with BMI emerging as the primary determinant [25]. Conversely, Yu et al. reported that INSTI exposure was independently associated with an increased risk of hepatic steatosis [26].

In light of these considerations, the impact of specific ART classes on hepatic steatosis risk in WoWH remains uncertain, underscoring the need for larger, sex‐stratified prospective studies.

A unique aspect of our study was the assessment of visceral fat distribution via US, encompassing abdominal, pre‐peritoneal, subcutaneous, epicardial and perirenal compartments. Although magnetic resonance imaging (MRI), Dual‐energy X‐ray Absorptiometry (DXA) and bioelectrical impedance analysis (BIA) represent reference‐standard techniques [27, 28, 29], these modalities are frequently inaccessible, costly and in some cases require contrast administration or ionizing radiation. Despite these limitations and the absence of universally validated reference values, our US‐based findings demonstrated a significant association between hepatic steatosis and increased fat accumulation across multiple compartments, particularly subcutaneous, abdominal and epicardial fat. Neither hepatic steatosis nor epicardial fat correlated with carotid intima‐media thickness (cIMT) or with the probability of detecting atherosclerotic plaques.

Epicardial adipose tissue is recognized as a marker of MetS and hepatic steatosis, and as an independent cardiovascular risk factor [30, 31]. We investigated its association with cIMT, previously proposed as a marker of subclinical atherosclerosis in people with HIV [32]; however, no correlation was observed, likely because measured fat volumes remained below the reported risk thresholds [30].

Given the strong association between MetS and Grade 1 hepatic steatosis, we assessed whether NCEP–ATP III criteria could serve as a screening tool to identify hepatic steatosis. Although nearly all women with MetS had hepatic steatosis, many women with steatosis did not fulfil MetS criteria, indicating that the NCEP–ATP III framework alone is insufficient to reliably guide hepatic steatosis screening. Given the relatively wide confidence interval, likely reflecting the limited sample size, this finding should be interpreted with caution, as the true discriminatory performance may range from poor to acceptable. To our knowledge, no studies have addressed this question specifically in women with HIV, highlighting the need for HIV‐specific screening strategies for early detection and clinical intervention.

Our study has several limitations.

Firstly, its monocentric design and the voluntary nature of participation may have introduced self‐selection bias, as women more engaged in healthcare may have been more likely to accept enrolment, resulting in a relatively small sample size.

We acknowledge that several female‐specific factors that may influence the development of MetS and associated hepatic steatosis—including current or prior use of menopausal hormone therapy, reproductive history (e.g., parity, age at menarche and menopause), breastfeeding history, hormonal contraception use, history of gestational diabetes or hypertensive disorders of pregnancy, polycystic ovary syndrome and gynaecological surgical interventions such as hysterectomy and/or oophorectomy—were not included in the present analysis. However, the assessment of these variables was beyond the scope of the present study.

The limited sample size may also have reduced statistical power to detect associations of small‐to‐moderate effect size—particularly regarding the independent contribution of specific ART classes to hepatic steatosis risk—and findings related to cumulative ART exposure should therefore be interpreted with caution. The absence of a control group of HIV‐seronegative women further limits the generalizability of our findings. Larger, sex‐stratified prospective studies are needed to address these gaps.

Finally, US‐based assessment of hepatic steatosis and visceral fat depots does not represent the reference standard compared with MRI, DXA, CAP or transient elastography. US carries inherent limitations in reproducibility and sensitivity, particularly for the detection of mild steatosis and the precise quantification of visceral adiposity, and the absence of universal reference values further limits cross‐study comparability. Nevertheless, US represents a pragmatic, widely accessible, radiation‐free and cost‐effective modality for metabolic surveillance in routine HIV clinical care, where reference‐standard techniques are frequently unavailable or logistically unfeasible.

Despite these limitations, our findings carry relevant clinical implications for the management of WoWH in routine care. Given that MetS emerged as the sole independent predictor of hepatic steatosis in our cohort—with an adjusted OR exceeding 14—we recommend that systematic metabolic risk assessment be integrated into the routine follow‐up of women with HIV, irrespective of age, menopausal status or ART regimen.

Based on our data, abdominal US screening for hepatic steatosis appears particularly warranted in WoWH who fulfil MetS diagnostic criteria and could be reasonably proposed from the age of 40 onwards, when the convergence of HIV‐related metabolic burden and age‐associated metabolic deterioration has been associated with higher steatosis prevalence; in the presence of MASLD, annual US reassessment appears clinically justified. In the absence of MetS, a baseline assessment should nonetheless be offered, with subsequent reassessment every 3–5 years, or earlier in the presence of newly developed metabolic comorbidities or hepatic steatosis without identifiable metabolic risk factors. This strategy may represent a pragmatic and cost‐effective approach to hepatic surveillance in this population.

The high rate of incidental findings observed in our cohort (5/98, 5.1%) further supports the clinical value of systematic US evaluation. Future research should aim to expand the cohort to increase statistical power, incorporate a control group of HIV‐seronegative women to disentangle HIV‐specific from general metabolic contributions, and prospectively collect female‐specific variables and HIV‐related biomarkers—such as markers of chronic immune activation and systemic inflammation—that were beyond the scope of the present study.

## AUTHOR CONTRIBUTIONS

Conceptualization: Mariacristina Poliseno. Methodology: Mariacristina Poliseno, Antonio Cianciaruso. Formal analysis: Mariacristina Poliseno, Maurizio Zarcone. Investigation: Davide Capruzzi, Giuliana Metrangolo, Vittoria Lobalsamo, Alessandra Vigna, Felicia Dalitto, Marica Noviello. Resources: Mariacristina Poliseno, Francesco Di Gennaro, Annalisa Saracino. Data curation: Antonio Cianciaruso, Nicola Colavito. Writing—original draft preparation: Davide Capruzzi, Antonio Cianciaruso, Nicola Colavito. Writing—review and editing: Mariacristina Poliseno, Antonio Cianciaruso, Nicola Colavito. Visualization: Mariacristina Poliseno. Supervision: Mariacristina Poliseno. Project administration: Vincenzo Ostilio Palmieri, Francesco Di Gennaro, Annalisa Saracino. All authors have read and agreed to the published version of the manuscript.

## CONFLICT OF INTEREST STATEMENT

The authors declare no conflicts of interest.

## ETHICS STATEMENT

The study was conducted in accordance with the Declaration of Helsinki and was approved by the local Ethics Committee (approval number: 2249/CEI, 18 June 2025). Written informed consent was obtained from all participants prior to enrolment.

## Supporting information


**Table S1.** Laboratory metabolic assessment in women with HIV with and without liver steatosis.

**Table S2.** Anthropometric parameters of women with HIVS with and without liver steatosis.

## Data Availability

The data that support the findings of this study are available from the corresponding author upon reasonable request.
