# Supplementary material for: UltRaSoUnd‐guided evaLuAtion of metabolic health in a cohort of midlife women with HIV: The URSULA study
Source: HIV Med. 2026 Jul 28;27(10):1650–62. doi: 10.1111/hiv.70290 (PMC13631687; doi:10.1111/hiv.70290)
Supplement: Supplementary file 1 [file HIV-27-1650-s001.docx]

**Table S1.** Laboratory metabolic assessment in WoWH with and without liver steatosis.

| Variables | Overall  (N = 98) | WoWHIV w/o steatosis  (N = 43) | WoWHIV with steatosis  (N = 55) | p  value* |
| --- | --- | --- | --- | --- |
| Renal function, mean (± SD) |  |  |  |  |
| Creatinine (mg/dL) | 0.76 (±0.17) | 0.78 (±0.19) | 0.75 (±0.15) | 0.49 |
| eGFR (mL/min/m^2^) | 87.4 (±17.5) | 85.9 (±19.5) | 88.5 (±15.8) | 0.46 |
| Urate (mg/dL) | 4.49 (±1.15) | 4.24 (±1.08) | 4.69 (±1.17) | 0.06 |
| BUN (mg/dL) | 37.9 (±9.07) | 39.3 (±8.32) | 36.9 (±9.55) | 0.19 |
| Sodium (mmol/L) | 141 (±1.97) | 140 (±1.83) | 141 (±2.05) | 0.12 |
| Potassium (mmol/L) | 4.13 (±0.36) | 4.13 (±0.33) | 4.13 (±0.38) | 0.93 |
| Diabetes |  |  |  |  |
| Median blood sugar (mg/dL), mean (± SD) | 87.8 (±13.4) | 84.2 (±11.8) | 90.5 (±14.0) | **0.025** |
| Hb_A1c (%), mean (± SD) | 34.2 (±5.38) | 33.9 (±5.94) | 34.4 (±4.94) | 0.60 |
| Hb_A1c >42%, n (%) | 5 (5) | 2 (5) | 3 (5) | 0.88 |
| Insulin level (mIU/L), mean (± SD) | 13.6 (±10.7) | 10.5 (±4.40) | 16.1 (±13.3) | **0.016** |
| HOMA_IR, mean (± SD) | 11.4 (±31.7) | 16.2 (±47.6) | 7.61 (±3.45) | 0.08 |
| Liver function, mean (± SD) |  |  |  |  |
| AST (U/L) | 25.5 (±20.7) | 25.7 (±30.0) | 25.3 (±8.32) | 0.92 |
| ALT (U/L) | 25.8 (±29.2) | 26.0 (±41.6) | 25.6 (±13.8) | 0.94 |
| Ammonia (mol/L) | 37.6 (±15.1) | 35.4 (±15.3) | 39.3 (±14.9) | 0.20 |
| Total cholesterol (mg/dL) | 192 (±36.7) | 197 (±34.8) | 188 (±37.9) | 0.21 |
| HDL cholesterol(mg/dL) | 58.4 (±15.0) | 62.9 (±12.4) | 54.9 (±16.0) | **0.011** |
| LDL cholesterol(mg/dL) | 111 (±31.2) | 112 (±30.1) | 111 (±32.3) | 0.80 |
| Triglycerides (mg/dL) | 107 (±46.9) | 102 (±50.4) | 112 (±43.9) | 0.30 |
| Lipase(U/L), mean (± SD) | 38.8 (±10.8) | 39.0 (±10.8) | 38.6 (±10.8) | 0.83 |
| Amylase (U/L), mean (± SD) | 38.6 (±16.6) | 38.4 (±13.5) | 38.7 (±18.8) | 0.93 |
| Iron load, mean (± SD) |  |  |  |  |
| Iron (µg/dL) | 81.8 (±28.1) | 82.3 (±26.2) | 81.3 (±29.7) | 0.85 |
| Ferritin (ng/mL) | 57.8 (±46.3) | 51.9 (±42.0) | 62.4 (±49.3) | 0.27 |
| Transferrin (mg/dL) | 261 (±40.7) | 263 (±44.4) | 260 (±37.8) | 0.74 |
| Thyroid function, mean (± SD) |  |  |  |  |
| TSH (mIU/L) | 1.78 (±1.08) | 1.86 (±0.93) | 1.72 (±1.18) | 0.52 |
| FT4 (pmol/L) | 1.19 (±0.33) | 1.19 (±0.40) | 1.19 (±0.28) | 0.95 |
| FT3 (pmol/L) | 3.33 (±0.43) | 3.26 (±0.54) | 3.39 (±0.31) | 0.14 |
| C-reactive protein (mg/dL), mean (± SD) | 2.58 (±2.10) | 2.25 (±1.71) | 2.84 (±2.34) | 0.17 |
| D-dimer (ng/mL), mean (± SD) | 421 (±536) | 370 (±259) | 462 (±678) | 0.42 |

****Chi-square test/ Student’s t-test as appropriate***

*WoWH= women with HIV, SD= standard deviation, eGFR= estimated glomerular filtration rate, BUN= blood urea nitrogen, HOMA-IR= homeostasis model assessment of insulin resistance, AST= aspartate aminotransferase, ALT= alanine aminotransferase, HDL= high-density lipoprotein, LDL= low-density lipoprotein, TSH= thyroid stimulating hormone, FT4= free thyroxin, FT3= free triiodothyronine*
