# Supplementary material for: UltRaSoUnd‐guided evaLuAtion of metabolic health in a cohort of midlife women with HIV: The URSULA study
Source: HIV Med. 2026 Jul 28;27(10):1650–62. doi: 10.1111/hiv.70290 (PMC13631687; doi:10.1111/hiv.70290)
Supplement: Supplementary file 2 [file HIV-27-1650-s002.docx]

**Table S2.** Anthropometric parameters of WoWH with and without liver steatosis.

| Anthropometric parameters | Overall  (N = 98) | WoWHIV w/o steatosis  (N = 43) | WoWHIV with steatosis  (N = 55) | p  value* |
| --- | --- | --- | --- | --- |
| Weight (kg), mean (± SD) | 65.3 (± 13.6) | 62.9 (± 12.4) | 67.3 (± 14.4) | 0.12 |
| Height (m), mean (± SD) | 1.62 (± 0.07) | 1.62 (± 0.06) | 1.62 (± 0.08) | 0.77 |
| BMI (kg/m^2^), mean (± SD) | 24.8 (± 4.91) | 23.9 (± 4.55) | 25.6 (± 5.12) | 0.12 |
| BMI>25, n (%) | 39 (41) | 15 (33) | 24 (48) | 0.14 |
| Abdominal circumference (cm), mean (± SD) | 87.8 (± 11.4) | 84.4 (± 9.11) | 90.8 (± 12.5) | **0.014** |
| Abdominal circumference≥88cm, n (%) | 39 (46) | 13 (32) | 26 (59) | **0.013** |
| ABP ≥130/80 mmHg, n (%) | 43 (45) | 14 (33) | 29 (56) | **0.026** |
| Triglycerides >150 mg/dL, n (%) | 17 (17) | 8 (19) | 9 (16) | 0.78 |
| Fasting blood glucose ≥100 mg/dL, n (%) | 14 (14) | 2 (5) | 12 (22) | **0.016** |
| HDL-cholesterol <50 mg/dL, n (%) | 34 (35) | 7 (16) | 27 (49) | **0.001** |
| Framingham10-year risk score for HCHD, mean (± SD) | 0.02 (± 0.02) | 0.02 (± 0.02) | 0.02 (± 0.02) | 0.94 |
| MetS, n (%) | 24 (25) | 3 (7) | 21 (40) | **<0.001** |

****Chi-square test/ Student’s t-test as appropriate***

*WoWHIV= women with HIV, SD=standard deviation, BMI=body mass index,ABP=arterial blood pressure, HDL= high-density lipoprotein, HCHD= hard coronary heart disease,MetS= metabolic syndrome*
